# Assessment of “Spin” in the abstracts of systematic reviews in leading upper extremity surgery journals over the past 10 years: A cross-sectional methodological study

**DOI:** 10.1177/17585732261472066

**Published:** 2026-07-27

**Authors:** Oluwatoba T Balogun, Varun Jain, Praveen Sritharan, Aava Param, Ajay Shah, Darius Luke Lameire, Ujash Sheth

**Affiliations:** 1 Temerty Faculty of Medicine, 12366 University of Toronto, Toronto, Canada; 2 DeGroote School of Medicine, 3710 McMaster University, Hamilton, Canada; 3 Division of Orthopaedic Surgery, 12366 University of Toronto, Toronto, Canada; 4 Division of Orthopaedic Surgery, 71545 Sunnybrook Health Sciences Centre, Toronto, Canada

**Keywords:** Spin, orthopaedic surgery, upper extremity surgery, reporting bias, abstract misrepresentation

## Abstract

**Introduction:**

Practice guidelines in upper extremity surgery are heavily influenced by systematic reviews (SRs). SRs are prone to “spin”; misleading reporting that can distort interpretation, such as omitting individual study risk of bias when formulating study conclusions. This study aimed to assess the prevalence and types of spin employed in abstracts of SRs from leading upper extremity surgical journals.

**Methods:**

A cross-sectional analysis of SRs published between 2015 and 2025 was conducted across the three highest impact factor upper extremity journals. Included study abstracts were assessed for nine types of spin using a published framework by Yavchitz et al. (2016). Descriptive statistics were used to evaluate the prevalence of spin and its associations with study bibliometric characteristics.

**Results:**

Spin was found in 28/54 included articles (51.9%). 21/54 (38.9%) studies demonstrated more than one type of spin. The most common type of spin employed was type 9 (claims of benefit despite reporting bias) (27/54, 50.0%).

**Conclusion:**

Over 50% of SRs published in leading upper extremity surgical journals demonstrated at least one form of spin in their abstracts. Claiming benefits despite a high risk of bias was most commonly employed. These findings highlight the need for robust reporting frameworks to ensure SRs are interpreted effectively.

**Level of evidence:**

IV

## Introduction

The emphasis on evidence-based medicine has placed immense importance on the utility of medical literature to guide clinical decision making. The findings and conclusions drawn from clinical research have significantly influenced the way that medicine and surgery are practiced in the real world. Systematic reviews (SRs) are an example of a study methodology which can be utilized to consolidate evidence and formulate high-level conclusions on any research question.^1^ Despite their importance in the medical literature, they often rely on study authors to interpret findings of various studies (which between themselves could present with considerable heterogeneity in their respective methodologies and results) to report these high-level conclusions.

Spin refers to the practice of presenting study results in a way that makes them appear more favorable or significant than they may in fact be. Often, this is done unintentionally, largely due to oversight or an overzealous interpretation of research findings. It may involve various strategies, such as emphasizing statistically significant secondary outcomes when the primary outcome is not significant, extrapolating findings to a different outcome, or omitting risk of bias when reporting results in the conclusions of a study.^2^ Spin is prevalent across different types of biomedical research, including clinical trials, observational studies, and SRs, affecting the interpretation and dissemination of research findings.^2,3^ Yavchitz et al. (2016)^4^ outlined nine types of spin that are believed to be most common in literature, providing a framework for spin analysis within abstracts (Table 1).

**Table 1.** Nine types of spin in systematic reviews.

Type	Description
1	Conclusion contains recommendations for clinical practice not supported by the findings.
2	Title claims or suggests a beneficial effect of the experimental intervention not supported by the findings.
3	Selective reporting of or overemphasis on efficacy outcomes or analysis favoring the beneficial effect of the experimental intervention.
4	Conclusion claims safety based on non-statistically significant results with a wide confidence interval.
5	Conclusion claims the beneficial effect of the experimental treatment despite high risk of bias in primary studies.
6	Selective reporting of or overemphasis on harm outcomes or analysis favoring the safety of the experimental intervention.
7	Conclusion extrapolates the review's findings to a different intervention (i.e. claiming efficacy of one specific intervention although the review covers a class of several interventions).
8	Conclusion extrapolates the review's findings from a surrogate marker or a specific outcome to the global improvement of the disease.
9	Conclusion claims the beneficial effect of the experimental treatment despite reporting bias.

When surveying the journal reading habits of internal medicine physicians in a study conducted by Saint et al. (2000)^5^, 63% reported primarily reading a study's abstract and did not use the full text of these respective articles to make their own interpretations of the study's findings. Similarly, a randomized control trial of 300 medical oncologists conducted by Boutron et al. (2014)^6^ demonstrated that the presence of spin in a sample of abstracts was associated with participants subjectively misinterpreting an intervention as beneficial over a comparator. This underscores the importance of precise abstract reporting and conclusions that are accurately divulged from the study design to maintaining a high standard of care. In the field of upper extremity surgery which is among the more research-productive orthopedic subspecialties, this becomes even more important.^7,8^

In orthopedic surgery, treatment decisions often rely on the findings of randomized controlled trials and the summation of evidence in SRs and meta-analyses. Spin has been shown to be highly prevalent across smaller analyses of SRs related to specific upper extremity topics—occurring in one-third of studies on rotator cuff tears, 34% of studies on proximal humeral fractures, 54% of studies on tendon transfers in massive irreparable rotator cuff tears, 93% of studies evaluating subacromial balloon spacers in the treatment of massive irreparable rotator cuff tears, and in 100% of studies on superior capsular reconstruction.^9–13^ This raises concerns, as surgeons may rely heavily on abstracts when making clinical decisions, and the presence of spin may inadvertently influence clinical practice.

Though spin has been found to be frequently demonstrated in the abstracts of SRs across upper extremity surgery, it remains to be investigated whether this issue extends to SRs published in journals of the highest impact factor in this field based on Journal Citation Reports. With these SRs having the most outreach in the general orthopedic community, it is important to assess their standard of methodological rigor.

Ultimately, the purpose of this review is to identify spin in its various forms and its prevalence in SRs abstracts from leading English-based orthopedic surgery journals that report “positive” results (statistically significant differences favoring either the intervention or comparator groups) within their samples. Secondarily, we aim to analyze studies and test for an association between the prevalence of spin and study/bibliometric factors.

## Methods

This study employed a cross-sectional design using SR methodology to identify and evaluate the presence of spin in abstracts of SRs and meta-analyses published in high-impact journals within the field of upper extremity surgery. The review was conducted in accordance with the Cochrane Handbook for Systematic Reviews of Interventions and reported following the preferred reporting items for systematic reviews and meta-analyses (PRISMA) guidelines.^14^ A protocol, created by the authors prior to data collection, was followed a priori for the literature search, screening, data extraction, and spin assessment phases.

### Literature search and inclusion/exclusion criteria

A systematic search was performed in MEDLINE, Embase, and PubMED from 26 March 2015 to 26 March 2025. The complete search strategy is included as a supplementary document (S1).

Articles were eligible for inclusion if they met the following criteria: (1) published in one of the three top impact factor upper extremity and hand journals identified by 5-year Journal Citation Reports impact factor at the time of the search (April 2025): Journal of Shoulder and Elbow Surgery, Journal of Hand Surgery (American Volume), and Journal of Hand Surgery (European Volume); (2) published in English; (3) designed as a SR or meta-analysis; (4) comparative in nature, reporting at least one clinical outcome comparing intervention and comparator groups; and (5) reporting a statistically significant difference in at least one outcome in the abstract.

Articles were excluded if they met any of the following criteria: (1) not a SR or meta-analysis; (2) primary outcome was not clinical; (3) abstract did not report a statistically significant difference between groups; or (4) review focused solely on non-therapeutic or prognostic questions. The exclusion of non-therapeutic SRs was aligned with prior literature emphasizing that spin is most relevant in studies evaluating clinical outcomes of interventions.^4^

### Article screening

Two reviewers (OB and VJ) independently screened all titles/abstracts and subsequently the full text of eligible studies in duplicate. Disagreements at the full-text stage were resolved through consensus or adjudication by a senior author (PS). Agreement between reviewers was assessed using Cohen's kappa (κ) statistic, interpreted a priori as follows: κ ≥ 0.80 (almost perfect), 0.60–0.79 (substantial), 0.40–0.59 (moderate), 0.20–0.39 (fair), and <0.20 (slight agreement).

### Data extraction

Data were independently extracted by two reviewers (OB and VJ) with graduate-level training in health research methodology using a standardized spreadsheet (Google Sheets, Google LLC, Mountain View, CA) developed a priori. Prior to formal extraction, reviewers underwent a multi-step pilot training process to ensure inter-rater reliability including a pilot spin assessment of the first 10 abstracts to calibrate their evaluation of spin. Disagreements were resolved by a third reviewer (PS). Spin was assessed for each study based on the nine types described by Yavchitz et al. (2016),^4^ applied specifically to the abstract conclusions (Table 1). Studies with statistically significant claims in the abstract had their full-text cross-referenced to confirm whether the data represented in the results section were concordant with this interpretation.

Additional extracted study characteristics included: publication year, country of corresponding author, upper extremity subspecialty topic (e.g. shoulder, elbow, hand/wrist), intervention and comparator descriptions, total number of included studies or participants, whether a meta-analysis was conducted, level of evidence of included primary studies, and total and annual citation counts.

### Outcomes

The primary outcome was the presence of spin in the abstract conclusion of each included SR or meta-analysis. Articles containing two or more distinct types of spin were categorized as exhibiting “severe spin,” a threshold defined *a priori.* The prevalence of spin was compared between reviews based on study characteristics such as inclusion of randomized controlled trials (level I/II evidence) versus reviews limited to observational studies (level III/IV evidence).

### Statistical analysis

Statistical analyses were conducted using RStudio (Version 4.4.2). Descriptive statistics, including means, standard deviations, frequencies, and percentages, summarized study characteristics and spin prevalence. Associations between categorical variables were analyzed using chi-square tests (with post-hoc analysis if appropriate), while correlations between continuous and categorical variables were evaluated using Pearson's biserial correlation coefficient.

Sample size calculations determined that at least 84 studies were needed to achieve 80% power to detect an effect size of 0.15 with four independent variables at α = 0.05 for multivariate analysis. If the sample size fell below this threshold, only univariate analyses were performed, consistent with the protocol. Citation analysis was limited to articles published up to 2024 to allow at least 1 year for citation accrual.

## Results

### Study characteristics

**Figure 1.**
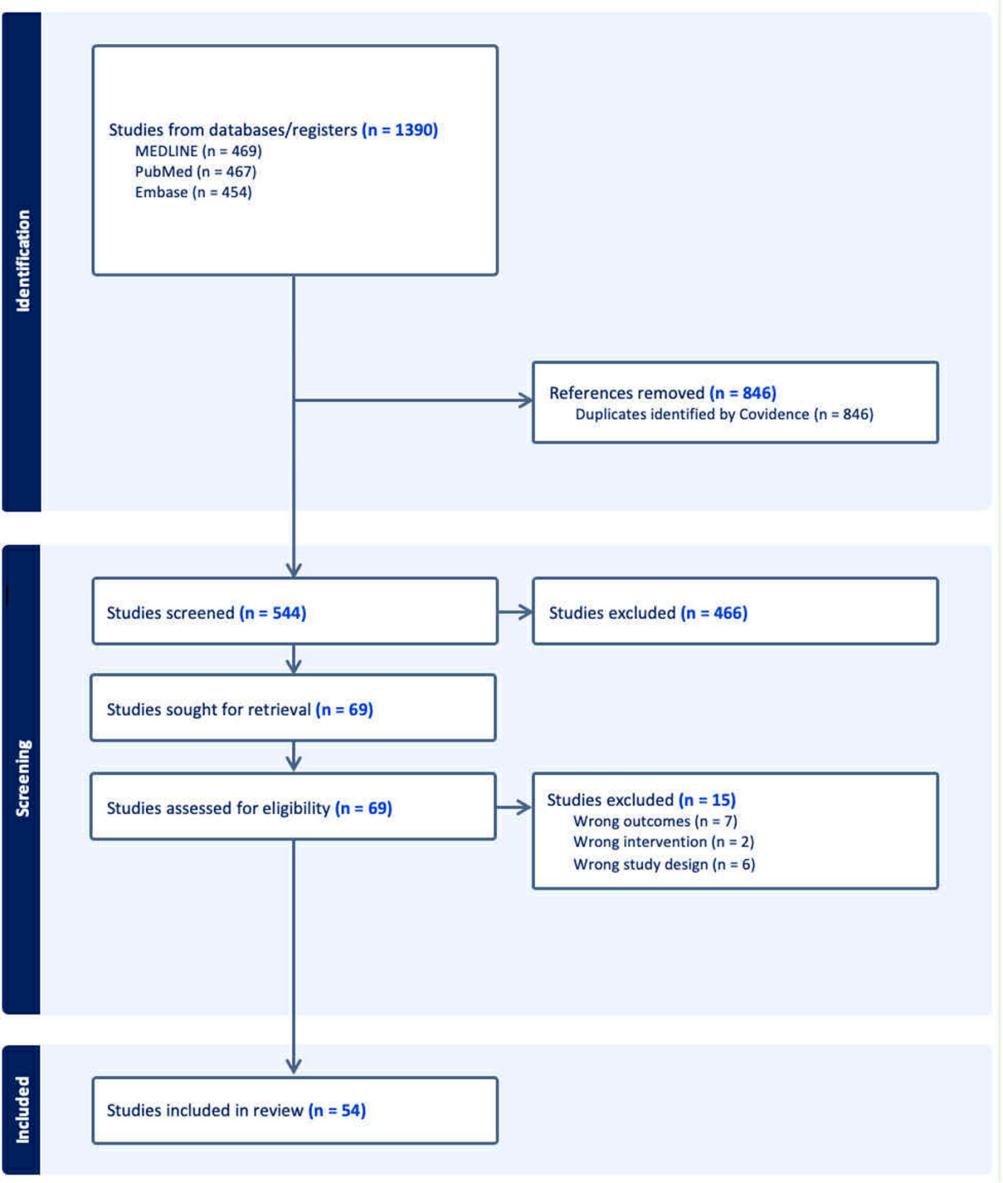
Preferred reporting items for systematic reviews and meta-analyses (PRISMA) flow diagram.

The initial search identified 1390 articles with 544 articles remaining after duplicates were removed. After a thorough title/abstract and full-text screening assessment, a total of 54 SRs were included for analysis (Figure 1).^15–68^ κ at title/abstract stage was 0.78 [95% CI 0.70 to 0.86] and at full-text stage was 0.91 [95% CI 0.80 to 1.00], indicating substantial agreement for the title/abstract stage and almost perfect agreement for the full-text stage. At the spin analysis stage, inter-rater reliability assessed with κ was found to be 0.76 [95% CI 0.67 to 0.85], depicting substantial agreement. Of the 54 included articles, 42/54 (77.8%) were published in the Journal of Shoulder and Elbow Surgery, 9/54 (16.7%) were published in the Journal of Hand Surgery (American Volume), and 3/54 (5.6%) were published in Journal of Hand Surgery (European Volume). Shoulder arthroplasty (28.8%), shoulder instability (15.4%), and wrist fractures (7.4%) were the most represented topics within the included SRs. The mean number of citations per publication was 29.3 (SD ± 40.4). Complete study characteristic data for the 54 included articles is provided in Supplementary File S2.

### Prevalence of spin

The abstracts of included SRs ranged from depicting 04 total types of spin (Table 1), with a median of 1 type. The prevalence of spin in the included articles was 28/54 (51.9%), and the prevalence of severe spin was 21/54 (38.9%). The three most common types of spin that were found in the included SRs include: type 9 “*Conclusion claims the beneficial effect of the experimental treatment despite reporting bias”* (27/54, 50.0%); type 5 “*Conclusion claims the beneficial effect of the experimental treatment despite high risk of bias in primary studies”* (22/54, 40.7%); and type 3 “*Selective reporting of or overemphasis on efficacy outcomes or analysis favoring the beneficial effect of the experimental intervention”* (2/54, 3.7%). Two types of spin were not found in any of the included articles: type 6 “*Selective reporting of or overemphasis on harm outcomes or analysis favoring the safety of the experimental intervention”* and type 7 “*Conclusion extrapolates the review's findings to a different intervention.”*

There was no statistically significant association between the presence of spin and number of citations per year nor total citations (*χ^2^* = 5.323, *p* = 0.15).

### Association of spin with level of evidence

**Figure 2.**
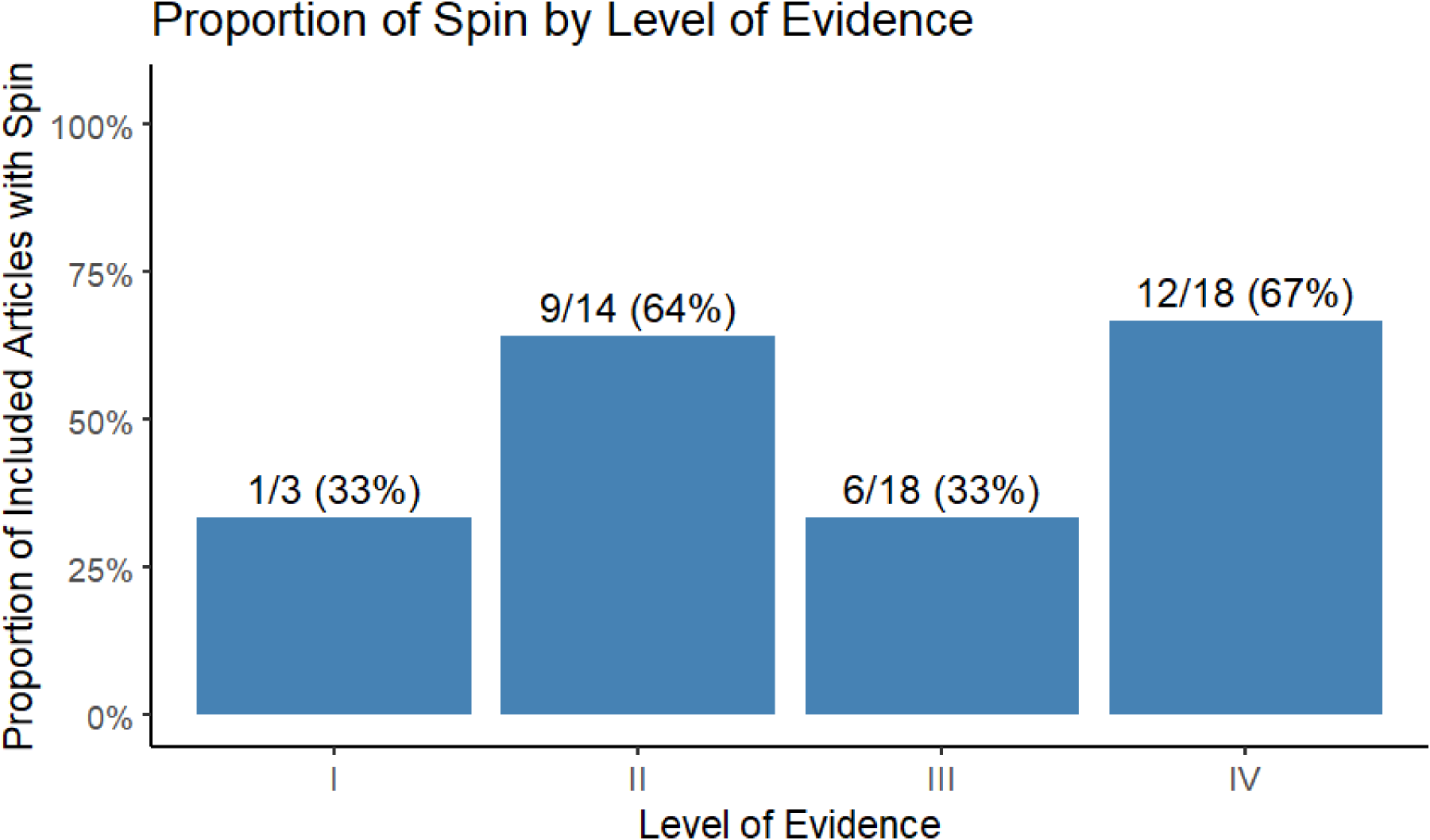
Proportion of spin in included articles stratified by level of evidence.

Of the included articles, three (5.6%) were classified as LOE I, 14 articles (25.9%) were classified as LOE II, 18 articles (33.3%) were classified as LOE III, and 18 articles (33.3%) were classified as LOE IV. The presence of spin was highest in LOE IV studies, with a 66.7% presence of spin (12/18 articles). This is followed by LOE II (9/14, 64.3%), and LOE I and IV (1/3, 33.3% and 6/18, 33.3%, respectively) (Figure 2). There was no statistically significant association with the presence of spin and the level of evidence of the included SR, nor was there a statistically significant difference in the presence of spin found in the abstracts of articles in the RCT SR group (LOE I and II) compared to the non-randomized study SR group (LOE III and IV) (*χ^2^* = 0.483, *p* = 0.49).

## Discussion

Our study found that spin was present in 51.9% of SRs published in the top three highest impact factor upper extremity and hand surgery journals, with 38.9% exhibiting what was defined in this study as severe spin. This demonstrates a substantial prevalence of misrepresentation of study findings in publication abstracts, even among work produced in high-impact journals.

The most common types of spin identified were type 9 “*asserting beneficial effects despite reporting bias”* (27/54, 50.0%), type 5 “*claiming benefit despite high risk of bias in primary studies”* (22/54, 40.7%), and type 3 “*selective overemphasis on efficacy outcomes”* (2/54, 3.7%). These trends closely mirror findings in other orthopedic subspecialties. For instance, trauma literature showed a 66% prevalence of spin, commonly due to emphasis on secondary endpoints or subgroup analyses, while lower extremity arthroplasty literature exhibited a 58.7% rate of spin, similarly driven by selective reporting.^69,70^

Notably, the most prevalent types of spin in our review centered on bias underreporting rather than overemphasis of statistical significance alone. This suggests a particular vulnerability in SRs where bias is either not adequately assessed or omitted from abstract conclusions. These findings reinforce prior work showing that up to 59% of SRs fail to consider risk of bias in their abstract or conclusions^71,72^ The PRISMA guidelines themselves which have extensive reporting guidelines for manuscripts only feature one recommendation for abstracts; therefore, there is minimal guidance governing reporting accuracy in study abstracts. Including risk of bias details directly in the abstract or accurately temporizing results in studies with high risk of bias may be helpful in avoiding misleading reporting. Taken together, this finding urges authors and journal reviewers to assess for this critical criterion of spin among upper extremity literature and orthopedic research broadly.

Our study found no statistically significant association between the presence of spin and journal bibliometrics, number of citations, or level of evidence. This points to a broad prevalence of spin and the inherent flaws of the publishing process across the selected journals and all types of SRs. Again, this finding supports a growing concern that spin reflects broader flaws in reporting culture and underscores the need for improved editorial standards, greater transparency in reporting, and targeted education for authors and peer reviewers. Moreover, given that clinical research—particularly in orthopedic surgery—is frequently misrepresented on news and social media outlets, it is incumbent upon the scientific community to act as a reliable and unbiased source of information.^73,74^ Incorporating structured assessments of risk of bias directly into abstract conclusions—especially in SRs or expanding guidelines like PRISMA for Abstracts to explicitly caution against spin may be sound strategies to decrease the prevalence of spin.

This study has several limitations. First, we restricted our analysis to English-language articles published in three high-impact upper extremity journals, which may limit generalizability to lower-impact or non-English journals. Second, although we used a validated classification system by Yavchitz et al. (2016)^4^ for identifying spin, the process of evaluating spin remains inherently subjective despite good inter-rater reliability. Third, we focused exclusively on reviews that reported statistically significant results, which may have introduced selection bias and potentially overestimated the prevalence of spin. Lastly, we were unable to include enough studies to conduct a multivariate analysis which could have provided more in-depth insights on the presence of spin.

Future work should prioritize the development and implementation of clear guidelines and reporting frameworks to ensure that abstracts published in the scientific literature contain minimal to no spin. Additionally, since this analysis focused specifically on spin in abstracts, future research should extend this work by developing a framework specific to the assessment of spin in full-text articles and by systematically examining full texts for the presence of spin. Though the Yavchitz et al. (2016)^4^ publication did recognize domains of spin that may be found in full-text articles, these did not undergo the same consensus agreement process among methodologist to determine the nine most severe forms of spin. The article explicitly stated that the framework presented was only relevant to abstracts of SRs and meta-analyses.^4^ Nonetheless, a similar consensus agreement process can be undertaken to focus on full-text articles

## Conclusion

Our findings demonstrate that approximately half (51.9%) of the SRs in the three highest impact upper extremity journals over the past 10 years demonstrate at least one form of spin in their abstracts. This was most commonly manifested by failure to incorporate risk of bias assessments in the conclusions of abstracts of SRs published in these journals. Authors must remain vigilant in their synthesis and reporting practices in their abstracts, while readers and reviewers must adopt a critical lens when appraising the literature. Promoting accuracy in the interpretation of evidence is essential to uphold the integrity of evidence-based surgical care.

## Supplemental Material

sj-docx-1-sel-10.1177_17585732261472066 - Supplemental material for Assessment of “Spin” in the abstracts of systematic reviews in leading upper extremity surgery journals over the past 10 years: A cross-sectional methodological study Supplemental material, sj-docx-1-sel-10.1177_17585732261472066 for Assessment of “Spin” in the abstracts of systematic reviews in leading upper extremity surgery journals over the past 10 years: A cross-sectional methodological study by Oluwatoba T Balogun, Varun Jain, Praveen Sritharan, Aava Param, Ajay Shah, Darius Luke Lameire and Ujash Sheth in Shoulder & Elbow

sj-xlsx-2-sel-10.1177_17585732261472066 - Supplemental material for Assessment of “Spin” in the abstracts of systematic reviews in leading upper extremity surgery journals over the past 10 years: A cross-sectional methodological study Supplemental material, sj-xlsx-2-sel-10.1177_17585732261472066 for Assessment of “Spin” in the abstracts of systematic reviews in leading upper extremity surgery journals over the past 10 years: A cross-sectional methodological study by Oluwatoba T Balogun, Varun Jain, Praveen Sritharan, Aava Param, Ajay Shah, Darius Luke Lameire and Ujash Sheth in Shoulder & Elbow

sj-docx-3-sel-10.1177_17585732261472066 - Supplemental material for Assessment of “Spin” in the abstracts of systematic reviews in leading upper extremity surgery journals over the past 10 years: A cross-sectional methodological study Supplemental material, sj-docx-3-sel-10.1177_17585732261472066 for Assessment of “Spin” in the abstracts of systematic reviews in leading upper extremity surgery journals over the past 10 years: A cross-sectional methodological study by Oluwatoba T Balogun, Varun Jain, Praveen Sritharan, Aava Param, Ajay Shah, Darius Luke Lameire and Ujash Sheth in Shoulder & Elbow

## References

[1] Uman LS . Systematic reviews and meta-analyses. J Can Acad Child Adolesc Psychiatry 2011; 20: 57–59. 21286370 PMC3024725

[2] Chiu K Grundy Q Bero L . Spin’ in published biomedical literature: A methodological systematic review. PLoS Biol 2017; 15: e2002173. 10.1371/journal.pbio.2002173 PMC5593172 28892482

[3] Boutron I Ravaud P . Misrepresentation and distortion of research in biomedical literature. Proc Natl Acad Sci USA 2018; 115: 2613–2619. 29531025 10.1073/pnas.1710755115 PMC5856510

[4] Yavchitz A Ravaud P Altman DG , et al. A new classification of spin in systematic reviews and meta-analyses was developed and ranked according to the severity. J Clin Epidemiol 2016; 75: 56–65. 26845744 10.1016/j.jclinepi.2016.01.020

[5] Saint S Christakis DA Saha S , et al. Journal reading habits of internists. J Gen Intern Med 2000; 15: 881–884. 11119185 10.1046/j.1525-1497.2000.00202.x PMC1495716

[6] Boutron I Altman D Hopewell S , et al. Impact of spin in the abstracts of articles reporting results of randomized controlled trials in the field of cancer: The SPIIN randomized controlled trial. J Clin Oncol 2014; 32: 4120–4126. 25403215 10.1200/JCO.2014.56.7503

[7] Mei X Zhu X Zhang T , et al. Worldwide productivity in the hand and wrist literature: A bibliometric analysis of four highly cited subspecialty journals. Int J Surg 2016; 28: 8–12. 26876956 10.1016/j.ijsu.2016.02.029

[8] Haimowitz S Veliky J Forrester LA , et al. Subspecialty selection impacts research productivity and faculty rank of academic orthopaedic surgeons. J Bone Joint Surg Am 2022; 104: e31. 10.2106/JBJS.21.00443 34793371

[9] Reddy AK Shepard S Ottwell R , et al. Over 30% of systematic reviews and meta-analyses focused on rotator cuff tear treatments contained spin in the abstract. Arthrosc - J Arthrosc Relat Surg 2021; 37: 2953–2959. 10.1016/j.arthro.2021.03.066 33887409

[10] Jones C Rulon Z Arthur W , et al. Evaluation of spin in the abstracts of systematic reviews and meta-analyses related to the treatment of proximal humeral fractures. J Shoulder Elbow Surg 2021; 30: 2197–2205. 33482369 10.1016/j.jse.2020.11.026

[11] Bashrum BS Hwang NM Thompson AA , et al. Evaluation of spin in systematic reviews on the use of tendon transfer for massive irreparable rotator cuff tears. J Shoulder Elbow Surg 2024; 33: e377–e383. 10.1016/j.jse.2023.10.036 38122887

[12] Fathi A Bashrum BS Kim MS , et al. Evaluation of spin in reviews of biodegradable balloon spacers for massive irreparable rotator cuff tears. J Shoulder Elbow Surg 2024; 33: e13–e20. 10.1016/j.jse.2023.07.033 37657596

[13] Kim MS Hasan LK Fathi A , et al. Evaluation of spin in systematic reviews and meta-analyses of superior capsular reconstruction. J Shoulder Elbow Surg 2022; 31: 1743–1750. 35472573 10.1016/j.jse.2022.03.015

[14] Page MJ McKenzie JE Bossuyt PM , et al. The PRISMA 2020 statement: An updated guideline for reporting systematic reviews. BMJ 2021; 372: n71. 10.1136/bmj.n71 PMC8005924 33782057

[15] Abdel Khalik H Lameire DL Leroux T , et al. Arthroscopic stabilization surgery for first-time anterior shoulder dislocations: A systematic review and meta-analysis. J Shoulder Elbow Surg 2024; 33: 1858–1872. 38430981 10.1016/j.jse.2024.01.037

[16] Ahmed AF Polisetty TS Wang C , et al. Higher return to sport and lower revision rates when performing arthroscopic Bankart repair with Remplissage for anterior shoulder instability with a Hill-Sachs lesion: A meta-analysis. J Shoulder Elbow Surg 2024; 33: 1836–1846. 38499236 10.1016/j.jse.2024.01.045

[17] Ajrawat P Dwyer T Almasri M , et al. Bone marrow stimulation decreases retear rates after primary arthroscopic rotator cuff repair: A systematic review and meta-analysis. J Shoulder Elbow Surg 2019; 28: 782–791. 30885313 10.1016/j.jse.2018.11.049

[18] Alkhatib N Abdullah ASA AlNouri M , et al. Short- and long-term outcomes in Bankart repair vs. conservative treatment for first-time anterior shoulder dislocation: A systematic review and meta-analysis of randomized controlled trials. J Shoulder Elbow Surg 2022; 31: 1751–1762. 35398165 10.1016/j.jse.2022.02.032

[19] Alnaeem H Aldekhayel S Kanevsky J , et al. A systematic review and meta-analysis examining the differences between nonsurgical management and percutaneous fixation of minimally and nondisplaced scaphoid fractures. J Hand Surg Am 2016; 41: 1135–1144.e1. 27707564 10.1016/j.jhsa.2016.08.023

[20] An VVG Sivakumar BS Phan K , et al. A systematic review and meta-analysis of clinical and patient-reported outcomes following two procedures for recurrent traumatic anterior instability of the shoulder: Latarjet procedure vs Bankart Repair. J Shoulder Elbow Surg 2016; 25: 853–863. 26809355 10.1016/j.jse.2015.11.001

[21] Blondin M Garner GL Hones KM , et al. Considerations for surgical treatment of neurogenic thoracic outlet syndrome: A meta-analysis of patient-reported outcomes. J Hand Surg 2023; 48: 585–594. 10.1016/j.jhsa.2023.03.005 37055338

[22] Colasanti CA Anil U Rodriguez K , et al. Optimal combination of arthroplasty type, fixation method, and postoperative rehabilitation protocol for complex proximal humerus fractures in the elderly: A network meta-analysis. J Shoulder Elbow Surg 2024; 33: e559–e574. 10.1016/j.jse.2024.03.040 38734127

[23] Daher M Boufadel P Fares MY , et al. Reverse versus anatomic total shoulder arthroplasty for glenohumeral osteoarthritis with intact cuff: A meta-analysis of clinical outcomes. J Shoulder Elbow Surg 2025; 34: 190–202. 39142432 10.1016/j.jse.2024.06.026

[24] Dasari SP Kerzner B Fortier LM , et al. Improved outcomes for proximal humerus fracture open reduction internal fixation augmented with a fibular allograft in elderly patients: A systematic review and meta-analysis. J Shoulder Elbow Surg 2022; 31: 884–894. 34906682 10.1016/j.jse.2021.11.004

[25] Davey MS Davey MG Hurley ET , et al. Subscapularis management during open Latarjet procedure: Does subscapularis split versus tenotomy matter? A systematic review and meta-analysis. J Shoulder Elbow Surg 2022; 31: 2169–2175. 35461979 10.1016/j.jse.2022.03.008

[26] Del Core MA Cutler HS Ahn J , et al. Systematic review and network meta-analysis of subscapularis management techniques in anatomic total shoulder arthroplasty. J Shoulder Elbow Surg 2021; 30: 1714–1724. 33096273 10.1016/j.jse.2020.09.027

[27] Dubin JA Mirkin Y Sax OC , et al. Core decompression is superior to nonoperative management for humeral head osteonecrosis: A systematic review. J Shoulder Elbow Surg 2023; 32: 2192–2200. 37268284 10.1016/j.jse.2023.04.024

[28] Erickson BJ Frank RM Harris JD , et al. The influence of humeral head inclination in reverse total shoulder arthroplasty: A systematic review. J Shoulder Elbow Surg 2015; 24: 988–993. 25725965 10.1016/j.jse.2015.01.001

[29] Gopal N Anil A Gopal M , et al. A comparison between volar locking plates and percutaneous pinning in the treatment of distal radius fractures: A systematic review. J Hand Surg Am 2025; 50: 265–273. 39387751 10.1016/j.jhsa.2024.08.016

[30] Head L Gencarelli JR Allen M , et al. Wrist ganglion treatment: Systematic review and meta-analysis. J Hand Surg 2015; 40: 546–553.e8. 10.1016/j.jhsa.2014.12.014 25708437

[31] Hohmann E Tetsworth K Glatt V . Corticosteroid injections for the treatment of lateral epicondylitis are superior to platelet-rich plasma at 1 month but platelet-rich plasma is more effective at 6 months: An updated systematic review and meta-analysis of level 1 and 2 studies. J Shoulder Elbow Surg 2023; 32: 1770–1783. 37247780 10.1016/j.jse.2023.04.018

[32] Hohmann E Glatt V Tetsworth K . Minimally invasive plating versus either open reduction and plate fixation or intramedullary nailing of humeral shaft fractures: A systematic review and meta-analysis of randomized controlled trials. J Shoulder Elbow Surg 2016; 25: 1634–1642. 27522336 10.1016/j.jse.2016.05.014

[33] Hones KM Hao KA Rakauskas TR , et al. Four-corner fusion versus proximal row carpectomy for scapholunate advanced collapse and scaphoid nonunion advanced collapse wrist: A systematic review and meta-analysis. J Hand Surg 2024; 49: 633–648. 10.1016/j.jhsa.2024.01.011 38416092

[34] Hossein Zadeh R Daliri M Sadeghi M , et al. Arthroscopic Bankart repair vs. Latarjet procedure for recurrent shoulder instability: A meta-analysis of clinical outcomes and complication rates in general and athletic populations. J Shoulder Elbow Surg 2024; 33: e652–e674. 10.1016/j.jse.2024.06.024 39151667

[35] Hurley ET Wickman J Crook BS , et al. Intramedullary nailing vs. open reduction–internal fixation for humeral shaft fractures: A meta-analysis of randomized controlled trials. J Shoulder Elbow Surg 2023; 32: 2567–2574. 37579941 10.1016/j.jse.2023.07.015

[36] Hurley ET Toale JP Davey MS , et al. Remplissage for anterior shoulder instability with Hill-Sachs lesions: A systematic review and meta-analysis. J Shoulder Elbow Surg 2020; 29: 2487–2494. 32650087 10.1016/j.jse.2020.06.021

[37] Hurley ET Fat DL Duigenan CM , et al. Biceps tenodesis versus labral repair for superior labrum anterior-to-posterior tears: A systematic review and meta-analysis. J Shoulder Elbow Surg 2018; 27: 1913–1919. 29803502 10.1016/j.jse.2018.04.011

[38] Jain NP Mannan SS Dharmarajan R , et al. Tuberosity healing after reverse shoulder arthroplasty for complex proximal humeral fractures in elderly patients—does it improve outcomes? A systematic review and meta-analysis. J Shoulder Elbow Surg 2019; 28: e78–e91. 10.1016/j.jse.2018.09.006 30593437

[39] Kim CH Lee DH Lee JS , et al. Arthrodesis versus ligament reconstruction and tendon interposition for thumb carpometacarpal joint arthritis: A systematic review and meta-analysis. J Hand Surg Am 2025; 50: 282–291. 39652035 10.1016/j.jhsa.2024.10.018

[40] Lode I Nordviste V Erichsen JL , et al. Operative versus nonoperative treatment of humeral shaft fractures: A systematic review and meta-analysis. J Shoulder Elbow Surg 2020; 29: 2495–2504. 32553853 10.1016/j.jse.2020.05.030

[41] Looney AM Day J Bodendorfer BM , et al. Operative vs. nonoperative treatment of distal biceps ruptures: A systematic review and meta-analysis. J Shoulder Elbow Surg 2022; 31: e169–e189. 10.1016/j.jse.2021.12.001 34999236

[42] Lu V Jegatheesan V Patel D , et al. Outcomes of acute vs. delayed reverse shoulder arthroplasty for proximal humerus fractures in the elderly: A systematic review and meta-analysis. J Shoulder Elbow Surg 2023; 32: 1728–1739. 37024039 10.1016/j.jse.2023.03.006

[43] Masud S Momtaz D Betsch M , et al. A comprehensive comparison and evaluation of surgical techniques for anterior shoulder instability: A Bayesian network meta-analysis. J Shoulder Elbow Surg 2023; 32: e531–e547. 10.1016/j.jse.2023.07.004 37541334

[44] Matthewson G Kooner S Kwapisz A , et al. The effect of subscapularis repair on dislocation rates in reverse shoulder arthroplasty: A meta-analysis and systematic review. J Shoulder Elbow Surg 2019; 28: 989–997. 30827833 10.1016/j.jse.2018.11.069

[45] Meesters AML Assink N IJpma FFA . Functional outcome of 2-D- and 3-D-guided corrective forearm osteotomies: A systematic review. J Hand Surg Eur Vol 2024; 49: 843–851. 37747738 10.1177/17531934231201962 PMC11264531

[46] Mercurio M Castricini R Castioni D , et al. Better functional outcomes and a lower infection rate can be expected after superior capsular reconstruction in comparison with latissimus dorsi tendon transfer for massive, irreparable posterosuperior rotator cuff tears: A systematic review. J Shoulder Elbow Surg 2023; 32: 892–906. 36528222 10.1016/j.jse.2022.11.004

[47] Murray E Challoumas D Putti A , et al. Effectiveness of sodium hyaluronate and ADCON-T/N for the prevention of adhesions in hand flexor tendon surgery: A systematic review and meta-analysis. J Hand Surg 2022; 47: 896.e1–896.e20. 10.1016/j.jhsa.2021.07.012 34509314

[48] Onggo JR Nambiar M Onggo JD , et al. Improved functional outcome and tuberosity healing in patients treated with fracture stems than nonfracture stems during shoulder arthroplasty for proximal humeral fracture: A meta-analysis and systematic review. J Shoulder Elbow Surg 2021; 30: 695–705. 33157239 10.1016/j.jse.2020.09.044

[49] O’Sullivan J Lädermann A Parsons BO , et al. A systematic review of tuberosity healing and outcomes following reverse shoulder arthroplasty for fracture according to humeral inclination of the prosthesis. J Shoulder Elbow Surg 2020; 29: 1938–1949. 32815808 10.1016/j.jse.2020.03.032

[50] Paras T Raines B Kohut K , et al. Clinical outcomes of reverse total shoulder arthroplasty for elective indications versus acute 3- and 4-part proximal humeral fractures: A systematic review and meta-analysis. J Shoulder Elbow Surg 2022; 31: e14–e21. 10.1016/j.jse.2021.07.014 34454040

[51] Peng F Liu YX Wan ZY . Percutaneous pinning versus volar locking plate internal fixation for unstable distal radius fractures: A meta-analysis. J Hand Surg Eur Vol 2018; 43: 158–167. 29064312 10.1177/1753193417735810

[52] Peng Y Li F Ding Y , et al. Comparison of the effects of platelet-rich plasma and corticosteroid injection in rotator cuff disease treatment: A systematic review and meta-analysis. J Shoulder Elbow Surg 2023; 32: 1303–1313. 36868297 10.1016/j.jse.2023.01.037

[53] Piper CC Hughes AJ Ma Y , et al. Operative versus nonoperative treatment for the management of full-thickness rotator cuff tears: A systematic review and meta-analysis. J Shoulder Elbow Surg 2018; 27: 572–576. 29169957 10.1016/j.jse.2017.09.032

[54] Rodrigues-Lopes R Silva F Torres J . Periprosthetic shoulder infection management: One-stage should be the way: A systematic review and meta-analysis. J Shoulder Elbow Surg 2024; 33: 722–737. 37839627 10.1016/j.jse.2023.09.007

[55] Rossi LA Tanoira I Ranalletta M , et al. Cemented vs. uncemented reverse shoulder arthroplasty for proximal humeral fractures: A systematic review and meta-analysis. J Shoulder Elbow Surg 2022; 31: e101–e119. 10.1016/j.jse.2021.10.011 34737086

[56] Schoell K Crabb R Simpson E , et al. Preoperative corticosteroid injections are associated with a higher periprosthetic infection rate following primary total shoulder arthroplasty: A systematic review and meta-analysis. J Shoulder Elbow Surg 2024; 33: 2734–2742. 39002882 10.1016/j.jse.2024.05.025

[57] Shekouhi R Ahmed SH Mattia A , et al. Single versus double fascicular transfer for brachial plexus injuries: A systematic review and meta-analysis with meta-regression. J Hand Surg Eur Vol 2025; 50: 1091–1099. 39340257 10.1177/17531934241281187

[58] Shihab Z Sivakumar B Graham D , et al. Outcomes of arthroscopic-assisted distal radius fracture volar plating: A meta-analysis. J Shoulder Elbow Surg 2022; 47: 330–340.e1. 10.1016/j.jhsa.2021.11.025 35168831

[59] Shim JW Lee JS Park YB , et al. The effect of leucocyte concentration of platelet-rich plasma on outcomes in patients with lateral epicondylitis: A systematic review and meta-analysis. J Shoulder Elbow Surg 2022; 31: 634–645. 34861405 10.1016/j.jse.2021.10.036

[60] Shukla DR McAnany S Kim J , et al. Hemiarthroplasty versus reverse shoulder arthroplasty for treatment of proximal humeral fractures: A meta-analysis. J Shoulder Elbow Surg 2016; 25: 330–340. 26644230 10.1016/j.jse.2015.08.030

[61] Sullivan MA Adkinson JM . A systematic review and comparison of outcomes following simple syndactyly reconstruction with skin grafts or a dorsal metacarpal advancement flap. J Hand Surg 2017; 42: 34–40.e6. 10.1016/j.jhsa.2016.11.006 28052826

[62] Tan TK Tan P Wang K , et al. Effect of tranexamic acid on shoulder surgery: An updated meta-analysis of randomized studies. J Shoulder Elbow Surg 2024; 33: e97–e108. 10.1016/j.jse.2023.09.024 37890768

[63] Tansey PJ Yetter TR Somerson JS . Operative and nonoperative treatment of periprosthetic humerus fractures after shoulder arthroplasty: A systematic review and meta-analysis. J Shoulder Elbow Surg 2024; 33: e629–e636. 10.1016/j.jse.2024.04.009 38838842

[64] Xiao M Money AJ Pullen WM , et al. Outcomes after resection arthroplasty versus permanent antibiotic spacer for salvage treatment of shoulder periprosthetic joint infections: A systematic review and meta-analysis. J Shoulder Elbow Surg 2022; 31: 668–679. 34774777 10.1016/j.jse.2021.10.016

[65] Xiao M Cohen SA Cheung EV , et al. Pain management in shoulder arthroplasty: A systematic review and network meta-analysis of randomized controlled trials. J Shoulder Elbow Surg 2021; 30: 2638–2647. 34284094 10.1016/j.jse.2021.06.008

[66] Xu H Huang X Guo Z , et al. Outcome of surgical repair and rehabilitation of flexor tendon injuries in zone II of the hand: Systematic review and meta-analysis. J Hand Surg 2023; 48: 407.e1–407.e11. 10.1016/j.jhsa.2021.11.013 35131113

[67] Zhao D Han YH Pan KE , et al. The clinical efficacy of leukocyte-poor platelet-rich plasma in arthroscopic rotator cuff repair: A meta-analysis of randomized controlled trials. J Shoulder Elbow Surg 2021; 30: 918–928. 33220417 10.1016/j.jse.2020.10.014

[68] Zhu XM Leroux T Ben-David E , et al. A meta-analysis of level I evidence comparing tenotomy vs tenodesis in the management of long head of biceps pathology. J Shoulder Elbow Surg 2021; 30: 961–968. 33607334 10.1016/j.jse.2021.02.002

[69] Shepard S Checketts J Eash C , et al. Evaluation of spin in the abstracts of orthopedic trauma literature: A cross-sectional review. Injury 2021; 52: 1709–1714. 34020782 10.1016/j.injury.2021.04.060

[70] Checketts JX Riddle J Zaaza Z , et al. An evaluation of spin in lower extremity joint trials. J Arthroplasty 2019; 34: 1008–1012. 30733070 10.1016/j.arth.2019.01.016

[71] Hopewell S Boutron I Altman DG , et al. Incorporation of assessments of risk of bias of primary studies in systematic reviews of randomised trials: A cross-sectional study. BMJ Open 2013; 3: e003342. 10.1136/bmjopen-2013-003342 PMC3753473 23975265

[72] Katikireddi SV Egan M Petticrew M . How do systematic reviews incorporate risk of bias assessments into the synthesis of evidence? A methodological study. J Epidemiol Community Health 2015; 69: 189–195. 25481532 10.1136/jech-2014-204711 PMC4316857

[73] Bethell MA Anastasio AT Adu-Kwarteng K , et al. Analyzing the quality, reliability, and educational value of ACL rehabilitation exercises on TikTok: A cross-sectional study. Orthop J Sports Med 2023; 11: 23259671231218668. 38145222 10.1177/23259671231218668 PMC10748931

[74] Ekhtiari S Sun B Sidhu R , et al. Evidence versus frenzy in robotic total knee arthroplasty: A systematic review comparing news media claims to randomized controlled trial evidence. J Bone Joint Surg 2024; 106: 2384–2392. 39692716 10.2106/JBJS.24.00264

