# Supplementary material for: Assessment of “Spin” in the abstracts of systematic reviews in leading upper extremity surgery journals over the past 10 years: A cross-sectional methodological study
Source: Shoulder Elbow. 2026 Jul 27:17585732261472066. Online ahead of print. doi: 10.1177/17585732261472066 (PMC13407678; doi:10.1177/17585732261472066)
Supplement: sj-docx-1-sel-10.1177_17585732261472066 - Supplemental material for Assessment of “Spin” in the abstracts of systematic reviews in leading upper extremity surgery journals over the past 10 years: A cross-sectional methodological study [file sj-docx-1-sel-10.1177_17585732261472066.docx]

**Search Strategy**

PubMED March 26 2025

(("Journal of Shoulder and Elbow Surgery"[Journal]) OR ("Journal of Hand Surgery – American Volume"[Journal])) OR ("Journal of Hand Surgery – European Volume"[Journal])

*Filter publication dates between March 26, 2015 and March 26, 2025

—--

Ovid MEDLINE(R) ALL <1946 to March 27, 2025>

1 "journal of shoulder and elbow surgery".jn.

2 "journal of hand surgery – american volume".jn.

3 "journal of hand surgery – european volume".jn.

4 1 or 2 or 3

5 systematic review.mp. or "Systematic Review"/

6 meta-analysis.mp. or Meta-Analysis/

7 5 or 6

8 4 and 7

9 limit 8 to yr="2015 -Current"

—--

Embase <1974 to 2024 April 18>

1 "journal of shoulder and elbow surgery".jn.

2 "journal of hand surgery – american volume".jn.

3 "journal of hand therapy".jn.

4 1 or 2 or 3

5 systematic review.mp. or "systematic review"/

6 (meta-analysis or meta analysis).mp. or meta analysis/

7 5 or 6

8 4 and 7

9 limit 8 to yr="2015 -Current"
